# Transcriptomic Analysis of the Mechanisms for Alleviating Psoriatic Dermatitis Using Taodan Granules in an Imiquimod-Induced Psoriasis-like Mouse Model

**DOI:** 10.3389/fphar.2021.632414

**Published:** 2021-04-14

**Authors:** Le Kuai, Ying Luo, Keshen Qu, Yi Ru, Yue Luo, Xiaojie Ding, Meng Xing, Liu Liu, Xiaoying Sun, Xin Li, Bin Li

**Affiliations:** ^1^ Department of Dermatology, Yueyang Hospital of Integrated Traditional Chinese and Western Medicine, Shanghai University of Traditional Chinese Medicine, Shanghai, China; ^2^ Institute of Dermatology, Shanghai Academy of Traditional Chinese Medicine, Shanghai, China; ^3^ Department of Traditional Chinese Surgery, Longhua Hospital Shanghai University of Traditional Chinese Medicine, Shanghai, China; ^4^ Department of Dermatology, Shaanxi Hospital of Traditional Chinese Medicine, Xi`an, China; ^5^ Shanghai Dermatology Hospital, Tongji University, Shanghai, China

**Keywords:** psoriasis, taodan granules, RNA sequencing analysis, Chinese medicine, transcriptomic analysis

## Abstract

Taodan granules (TDGs) are clinically efficacious for treating psoriasis, buttheir specific mechanisms of action are unclear. In this study, we determined the concentrations of tanshinone IIA and curcumol using high-performance liquid chromatography (HPLC) to establish quality control parameters for assessing the mechanism of TDGs in treating psoriasis. Thereafter, a mouse model of psoriasis was treated with TDGs. TDGs attenuated imiquimod-induced typical erythema, scales, and thickening of the back and ear lesions in the psoriatic mouse model. Furthermore, PCNA and Ki67-positive cells were reduced in the *epidermis* of psoriatic lesions following TDG treatment. Finally, the sequencing results were verified using a multitude of methods, and the mechanism of action of TDGs against psoriasis was found to be via the upregulation of metabolic signaling pathways such as the Gly-Ser-Thr axis, the downregulation of immune and inflammatory pathways, and the decrease in Rac2 and Arhgdib concentrations. Overall, this study clarified the mechanism of TDG treatment for psoriasis and provided evidence for its clinical application.

## Background

Psoriasis is a common chronic refractory skin disease, characterized by epidermal hyperkeratosis, angiogenesis, and inflammatory reactions (Armstrong et al., 2020). The prevalence of psoriasis is increasing, ranging from 0.51 to 11.43% in adults, and a cross-sectional study revealed that psoriasis affects approximately 3% of the population and 7.4 million adults in the United States (Rachakonda et al., 2014; Michalek et al., 2017). Furthermore, the disease is associated with a significant economic burden, while the all-cause healthcare cost of each patient with psoriasis per year is generally $12,523 (Al Sawah et al., 2017). Psoriasis also has a serious negative impact on work productivity and health-related quality of life (Lopes et al., 2019). Psoriasis is a systemic disease that not only involves the skin, but is also associated with comorbidities, including cardiovascular disease, chronic obstructive pulmonary disease, diabetes, obesity, and hyperuricemia (Miele et al., 2009; Li et al., 2015; Yuan et al., 2019; Kovitwanichkanont et al., 2020). Systemic inflammation and oxidative stress in psoriasis are closely related to a variety of complications associated with a conspicuously high risk of death in patients with conditions such as kidney disease and liver disease (Dhana et al., 2019). Inflammation in psoriasis can lead to renal dysfunction by upregulating NADPH oxidases as well as inducible nitric oxide synthase (Al-Harbi et al., 2017a), and it has also been confirmed to induce hepatic inflammation, resulting in protein and lipid metabolism disorders through interleukin (IL)-17 RC/NF-κB signaling (Al-Harbi et al., 2017b). Additionally, the role of psoriatic inflammation enhances allergic airway inflammation via IL-23/signal transducer and activator of transcription 3 (STAT3) signaling in a murine model (Nadeem et al., 2017). Western medicine has made great progress in systematic therapies for psoriasis, such as methotrexate, cyclosporin, retinoic acid, and biological agents (Armstrong et al., 2020). Nonetheless, adverse reactions and contraindications have limited the wide application of these drugs (Sparks et al., 2020; Williams et al., 2020). Currently, Chinese medicine (CM) has become a global alternative medicine that has been gradually adopted for the treatment of psoriasis (Parker et al., 2017).

Taodan granules (TDGs) are composed of *Salvia miltiorrhiza Bunge*, *Curcuma aeruginosa Roxb.*, *Astragalus mongholicus Bunge*, *Glycyrrhiza inflata Batalin* and *Angelica sinensis (Oliv.) Diels*, *Conioselinum anthriscoides “Chuanxiong”*, *Prunus persica (L.) Batsch*, *Cyathula officinalis K. C. Kuan*, and *Smilax china L.*. Our previous clinical studies demonstrated that TDGs were satisfactory for the treatment of mild-to-moderate psoriasis vulgaris, and the reduction of IL-2, IL-4, and IL-6, together with the secretion of neuropeptides in the peripheral blood of patients with psoriasis was observed after TDG treatment for one month; the psoriasis area and severity index (PASI) score improved by 76.65% (Fan et al., 2006a; Fan et al., 2006b). Recently, Ru et al. conducted a high-quality randomized controlled trial to evaluate the clinical efficacy, safety, and recurrence rate of TDGs for psoriasis blood stasis syndrome (Ru et al., 2019). Although TDGs have a positive curative effect on psoriasis without significant AEs, the relative mechanism of action remains elusive.

RNA sequencing (RNA-seq), a progressive technique, is practical for identifying numerous genes regulated by specific medications (Liu et al., 2020). In this study, we used an imiquimod (IMQ)-induced psoriasis-like mouse model to examine the mechanism of action of TDGs against psoriasis. We used RNA-seq to analyze skin lesion samples with and without TDGs treatment, screened for the upregulated and downregulated genes 12°days after the therapy, conducted a bioinformatics study, and verified the aforementioned observations.

## Materials and Methods

### Pharmaceutical Composition of Taodan granule

The TDGs comprised nine Chinese herbs. These herbs (Supplementary Table S1) were authenticated by a pharmacognosist of the Yueyang Hospital of Integrated Traditional Chinese and Western Medicine, Shanghai University of Traditional Chinese Medicine, in accordance with standard protocols.

### High-Performance Liquid Chromatography

The extraction and HPLC analysis of the TDGs were performed using the following methods: 1) In line with standard methods of the Chinese Pharmacopeia, all crude drugs listed in Supplementary Table S1 were boiled and kept at near-boiling temperature for 1 h and then filtered. 2) Another 1,650 ml of water was added to the filtrate, brought to a boil again, kept for 1 h, and then filtered. 3) Two extracts were combined to obtain 1 g/ml of crude drug. Tanshinone IIA (abs47000393, purity ≥98%) and curcumol (abs47005976, purity ≥98%) were examined in the crude drug by using an Agilent 1,200 series HPLC and a ZORBAX SB-C18 chromatographic column (4.6 mm × 250 mm, 5 µm) therewith. The detection chromatographic conditions were as follows: acetonitrile, 0.5% phosphoric aqueous acid solution (70:30, v/v); column temperature, 30°C; detection wavelength, 210 nm; and injection volume, 20 µl. Tanshinone IIA and curcumol are the main active ingredients of *Salvia miltiorrhiza Bunge* and *Curcuma eruginosa Roxb,* respectively. Hence, TDGs without *Salvia miltiorrhiza Bunge* and *Curcuma eruginosa Roxb.* were used as the negative controls.

### Animals

Male specific pathogen-free (SPF)–grade BALB/c mice, with a weight of 25 ± 3 g, were provided by the Shanghai Medical Experimental Animal Center (SCXK Shanghai 2013–0016, Shanghai, China). The mice were maintained in an environment with a temperature of 23 ± 2°C and a 16–8 h light-dark cycle, along with sterile water. The fodder was supplied by Shanghai Pu Lu Tong Biological Technology Co., Ltd. All procedures were reviewed and approved by the Ethics Committee of Yueyang Hospital affiliated to Shanghai University of Traditional Chinese Medicine (no. YYLAC-2020–078–1).

### Plant Material and Drugs

The TDGs were acquired from the Pharmacy Department of the Yueyang Hospital of Integrated Traditional Chinese and Western Medicine, Shanghai University of Traditional Chinese Medicine, and stored at 4°C. Petroleum jelly was obtained from Nanchang Baiyun Pharmaceutical Co., Ltd (Jiangxi, China, Drug approval No. F20050006). IMQ cream was obtained from Sichuan Mingxin Pharmaceutical Co., Ltd (Sichuan, China, Drug approval No. H20030128).

### Experimental Grouping and Mice Model

Mice were randomly divided into three groups after the back hair was clipped (2 × 2 cm^2^).

a. Control group: ears and back were treated with 62.5 mg petroleum jelly.
b. IMQ group: ears and back were treated with 62.5 mg of 5% IMQ cream for 6 h, followed by intragastric administration of 1.8 g/kg 0.9% NaCl solution.
c. IMQ + TDG group: ears and back were treated with 62.5 mg of 5% IMQ cream for 6 h, followed by intragastric administration of 1.8 g/kg TDGs.

All treatments were executed from the date of applying IMQ cream (day 0), once a day for 12 days. The PASI score was used to determine the severity of skin inflammation on the backs and ears of the mice. Each parameter of the score ranged from 0 to 4, representing the order of severity. The mice were fasted before collecting specimens, but they could drink water for 12 h. Mice were euthanized by inhalation of CO_2_ on day 12. Lesions of the back and ear were collected for reserves.

### mRNA Sequencing Analysis

#### Sequencing Method

On day 12, the mice were euthanized. The back lesions were homogenized using TRIzol reagent (Ambion). Total RNA was extracted from the samples, and the DNA was digested with DNase. Magnetic beads with oligo (dT) enriched the mRNA. After adding the interrupting reagent, the mRNA was broken into short fragments, used as a template, and synthesized as one-strand cDNA with six base random primers. Next, a double-strand reaction system was prepared to synthesize double-stranded cDNA, which was purified using the kit. The purified double-stranded cDNA was subjected to terminal repair, with an A-tail connecting the sequencing connector. The fragment size was selected, and PCR amplification was performed. The library qualified with an Agilent 2,100 Bioanalyzer was established, and Illumina HiSeq™ 2,500 was used for sequencing to produce 125–150 bp double-ended data. RNA-seq analysis was performed by Shanghai OE Biotechnology Co., Ltd.

#### Gene Expression Analysis

Trimmomatic software (version 0.36) preprocessed the quality of the original data, and the number of reads during the entire quality control process was statistically summarized. Hisat2 software (version 2.2.1.0) was used to align CleanReads with the specified reference genome. We applied the known reference gene sequences and annotation files as the database, and the sequence similarity alignment method was adopted to identify the expression abundance of each protein-coding gene in each sample. The htseq-count software (version 0.9.1) was used to obtain the number of reads, compared with the protein-coding genes in each sample. The fragments per kilobase million (FPKM) values, representing the relative gene expression abundance, were calculated using the Cufflinks software (version 2.2.1).

#### Analysis of Differentially Expressed Genes

Standardization disposal was performed using DESeq software (version 1.18.0) for the gene count of each sample (BaseMean value was used to estimate expression). The multiples were calculated, and the negative binomial distribution test method (NB) was used to verify the significance of the difference in the number of reads. Finally, the different protein-coding genes were screened according to the results of fold changes and the significance of differences.

#### Gene Ontology and Kyoto Encyclopedia of Genes and Genomes Analysis

After the DEGs were obtained, the significance of the GO and KEGG analyses was analyzed using DAVID (https://david.ncifcrf.gov/). A *p*-value of <0.05 was considered as the standard for statistically significant differences. The list of target genes was set for “*Homo sapiens*”.

### Reverse Transcription Polymerase Chain Reactio

On day 12, the mice in each group were euthanized by CO_2_ inhalation. The middle portion of the skin lesions was measured. Total RNA was extracted using the TRIzol reagent kit. Afterward, the Reverse Transcription System First Strand cDNA Synthesis Kit was used with 20.0 a reaction volume. Real-time fluorescent PCR was used for the RT-PCR. This specific method was consistent with previous experiments (Kuai et al., 2018). The primer sequences are shown in Supplementary Table S2.

### Enzyme-Linked Immunosorbent Assay

On day 12, the lesions were collected from the mice for ELISA. The levels of chemokine (C-X-C motif) ligand 13 (CXCL13), IL-17a, tumor necrosis factor-alpha (TNF-α), sarcosine dehydrogenase (Sardh), and phosphoglycerate mutase 2 (Pgam2) protein expression were detected by applying a CXCL13 Mouse ELISA kit (ab212167, Abcam), an IL-17 Mouse ELISA kit (ab100702, Abcam), TNF-α Mouse ELISA kit (ab208348, Abcam), a Sardh Mouse ELISA kit (ABIN5521597), and a Pgam2 Mouse ELISA kit (ABIN5525256), respectively. Antibodies against the Vav 1 oncogene (Vav1) (ABIN6256653), LYN proto-oncogene, Src family tyrosine kinase (Lyn) (ABIN6256754), hematopoietic cell kinase (Hck) (ABIN3184982), protein kinase C, beta (Prkcb) (ABIN3032235), cystathionine beta-synthase (CBS) (ABIN6260518), serine dehydratase-like (Sdsl) (ABIN1092123), glycine N-methyltransferase (GNMT) (ABIN5700022), and Rho, GDP dissociation inhibitor (GDI) beta (Arhgdib) (ABIN5699138) from 4A Biotech Co. Ltd. were used for ELISA.

### Hematoxylin-Eosin Solution and Immunohistochemistry

The mice were euthanized by inhalation of CO_2_ on day 12. The center of the lesions were fixed with 4% formalin solution and stained with H&E solution, followed by immunohistochemistry (IHC). The quantitative methods for epidermal thickness together with the positive cell rate have been described in previous studies (Luo et al., 2019; Kuai et al., 2020).

The following antibodies were used for immunohistochemistry: anti-Ki-67 antibody (1:50, ab16667, Abcam), anti-PCNA antibody (1:6,400, ab29, Abcam), anti-NF-κB p105/p50 antibody (1:60,000, ab32360, Abcam), and anti-RAS-related C3 botulinum substrate 2 (Rac2) antibody (1:50, ab2244, Abcam).

### Western Blotting

Rac2 and Arhgdib protein expression in the back lesions was measured by western blotting. Briefly, the mice were euthanized by inhalation of CO_2_ on day 12. The middle portion of the back lesions was immediately placed in liquid nitrogen and stored at −80°C. The cells were collected. RIPA lysis buffer (Beyotime, Shanghai, China) was used to extract the total protein. The BCA protein analysis kit (TB258438; Bio-Rad, United States) was used to determine protein concentration. The total protein (20 μg per sample) was separated and transferred to a polyvinylidene fluoride membrane. The antibodies for western blotting included anti-Rac2 antibodies (ab2244; Abcam), anti-Arhgdib antibodies (ab181252; Abcam), and β-actin (ab8226; Abcam), and incubated overnight at 4°C. Next, the samples were incubated with the secondary antibody (ab205719; Abcam) for 1 h at room temperature.

### Statistical Methods

The data were analyzed using SPSS 24.0 (IBM Corp., Armonk International Business Machines, New York, United States), and they are described as mean ± standard deviation (SD). A *t*-test was used to compare the two groups. Statistical significance was set at *p* < 0.05.

## Results

### High-Performance Liquid Chromatography Profiles of Tanshinone IIA and Curcumol in Taodan Granule

**FIGURE 1:**
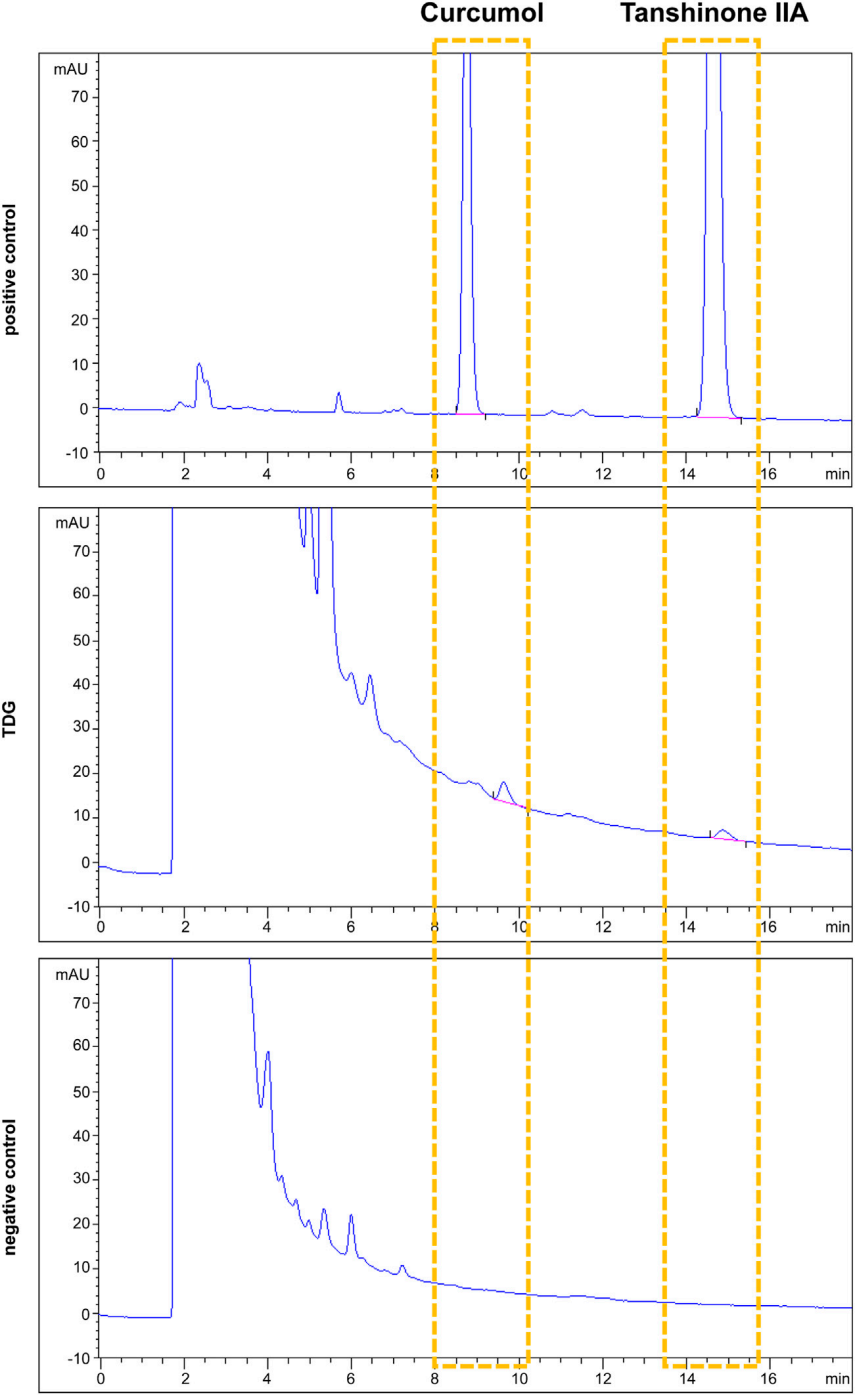
Tanshinone IIA and curcumol were adopted as quality controls for Taodan granule (TDG). Tanshinone IIA and curcumol are detected in both positive control and TDG samples but neither found in the negative control.

*Salvia miltiorrhiza Bunge* and *Curcuma aeruginosa Roxb.* are the two major components of the TDGs. Our previous study (Li et al., 2012) confirmed that tanshinone IIA, an active lipopolysaccharide of *Salvia miltiorrhiza Bunge*, could lead to cell cycle arrest and apoptosis in keratinocytes (KC) of the target cells in psoriasis. Tanshinone IIA has been shown to inhibit the increase in interferon sensitivity and upregulation of aberrant KC differentiation markers (Pedersen et al., 2012). On the other hand, curcumol is one of the main bioactive components of *Curcuma aeruginosa Roxb.*, and is generally used for the quality control of Chinese herbal compounds, which contains *Curcuma aeruginosa Roxb.* (Lei et al., 2019). Multiple studies have shown that curcumin can inhibit cell proliferation and migration in numerous proliferative diseases (Hashem et al., 2020; Huang et al., 2020; Li et al., 2020). In summary, we chose tanshinone IIA and curcumol to preliminarily establish quality control (Figure 1).

### Taodan Granules Relieved Back and ear Lesions, as Well as Suppressed Keratinocytes Proliferation in Imiquimod-Induced Psoriasis-like Mice

**FIGURE 2:**
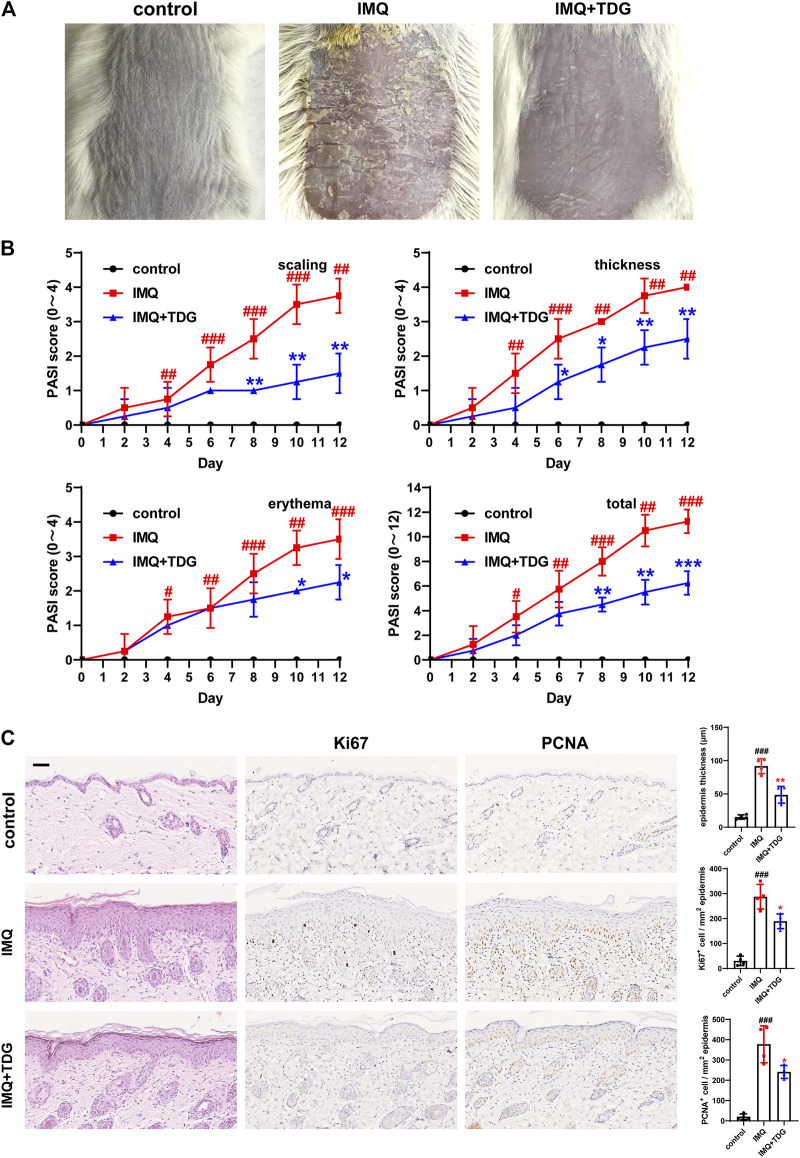
Taodan granules (TDGs) alleviated back lesions in imiquimod (IMQ)-induced psoriasis-like mice and decreased keratinocyte proliferation. **(A)** The appearance of back lesions in each group on day 12. **(B)** The psoriasis area severity index (PASI) score (0–4) with scales, thickness, erythema, and a total score. **(C)** Representative H&E sections of back lesions on day 12 (× 200) **(Left)**. Representative immunohistochemistry sections of Ki67 and PCNA nuclear staining (brown) of the back skin lesions (200×) **(Middle and Right)**. Quantification of *epidermis* thickness as well as Ki67^+^ and PCNA^+^ cells in back lesions. Scale bar: 100 µm. The data are expressed as mean ± SD. Four skin lesions from in group were included for analysis. ^#^ *p* < 0.05, ^##^ *p* < 0.01, ^###^ *p* < 0.001, compared with the control group. *p < 0.05, **p < 0.01, ***p < 0.001, compared with the IMQ group.

First, we established an animal model of psoriasis via IMQ induction. Compared with the control group, the IMQ group displayed typical psoriatic skin lesions with scales, erythema, and thickening accompanied by a conspicuous increase in the PASI scores (*p* < 0.05) (Figures 2A,B; Supplementary Figures S1A, B). Histologically, spinous layer hypertrophy, significantly thickened *epidermis* (back: *p* < 0.001; ear: *p* < 0.001), and obvious inflammation were observed in the IMQ-induced mice on day 12 (Figure 2C; Supplementary Figure S1C). These results revealed that the IMQ-induced mouse model was consistent with the characteristics of psoriasis.

Next, we determined whether TDGs could inhibit back and ear lesions *in vivo*. With TDG treatment, the skin lesions of IMQ-induced psoriasis-like mice were alleviated, and the PASI scores declined (*p* < 0.05) (Figures 2A,B; Supplementary Figures S1A, B). The most effective TDG dose was determined to be 1.8 g/kg (Supplementary Figure S2). On day 12, the TDG-treated mice demonstrated reductions in inflammatory cell infiltration and epidermal hyperplasia (back: *p* = 0.002; ear: *p* = 0.001) (Figure 2C; Supplementary Figure S1C).

We further predicted whether TDGs could prevent excessive KC proliferation in IMQ-induced mice. With TDG treatment, the lesions of the mice were validated by the significantly decreased the number of Ki67-positive cells (back: *p* = 0.014; ear: *p* = 0.014) as well as PCNA-positive cells (back: *p* = 0.03; ear: *p* = 0.019) (Figure 2C; Supplementary Figure S1C).

### mRNA Sequencing Analysis of Taodan Granule-Regulated Gene Expression in Imiquimod-Induced Psoriasis-Like Skin Lesions

#### Expression Analysis

We investigated the difference in gene expression with TDG treatment, RNA was extracted from the back tissues of the IMQ and IMQ + TDG groups for mRNA sequencing on day 12. Sequencing reports suggested that the total reads ranged from 47,337,442 to 50,470,212, and the rate of total mapped reads was between 94.97 and 95.79%. The boxplot for the FPKM values and the two-dimensional diagram of the principal component analysis are shown in Supplementary Figure S3.

#### Differentially Expressed Genes Following Taodan Granule Treatment

**FIGURE 3:**
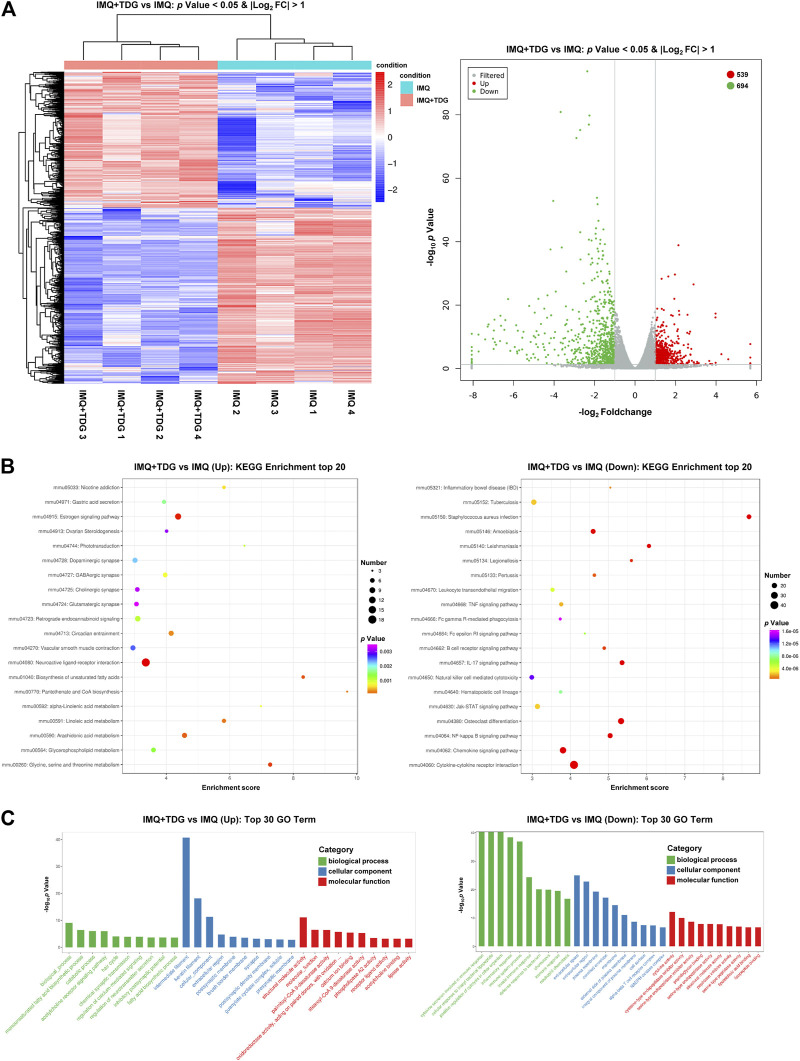
Differentially expressed genes (DEGs) induced after Taodan granule (TDG) treatment. **(A)** Cluster analysis of DEGs among samples and groups. The color of the heat map indicates the relative gene expression. The deeper red color indicates the higher gene expression, whereas the deeper blue color indicates the lower gene expression **(Left)**. The volcano map suggests the overall DEGs in the imiquimod (IMQ)+TDG group, compared with the IMQ group **(Right)**. **(B)** Enriched KEGG analysis of **up-(Left)** and **down-(Right)** regulated DEGs. **(C)** Enriched gene ontology analysis of **up-(Left)** and **down-(Right)** regulated DEGs.

DESeq software was used for differential expression analysis to identify candidate genes regulated by TDG treatment (1.18.0). |log_2_FoldChange| > 1 and *p*-value < 0.05 of the genes were judged for differential expression. A total of 1,233 DEGs were identified, of which 539 were upregulated and 694 were downregulated (Figure 3A). The 20 most significantly upregulated and downregulated DEGs after TDG treatment are demonstrated in Table 1. The mRNA levels for the 10 most significantly upregulated and downregulated DEGs were verified by RT-PCR, which were consistent with the sequencing results (Supplementary Figure S4).

**TABLE 1:** Top 20 upregulated and downregulated genes in the psoriatic skin of the mice models with or without Taodan granules (TDGs) (Genes with |log2FoldChange| > 1 and *p*-value < 0.05).

Gene ID	Gene name	Gene definition	LOG_2_∣FC∣	*p*-value	*Q*-value
Top 20 upregulated genes					
66,107	Wfdc21	WAP four-disulfide core domain 21	2.141,861,603	1.34E-39	1.15E-36
432,839	Gprin2	G protein regulated inducer of neurite outgrowth 2	1.969,102,658	2.22E-30	9.29E-28
108,151	Sema3d	Sema domain, immunoglobulin domain (ig), short basic domain, secreted, (semaphorin) 3D	1.643,954,426	8.96E-30	3.66E-27
16,008	Igfbp2	Insulin-like growth factor binding protein 2	1.311,271,087	4.79E-29	1.83E-26
406,220	Krt77	Keratin 77	2.889,566,113	2.87E-27	9.37E-25
230,613	Skint10	Selection and upkeep of intraepithelial T-cells 10	1.940,699,804	1.00E-22	2.61E-20
14,622	Gjb5	Gap junction protein, beta 5	1.0953,902	1.35E-19	2.34E-17
79,362	Bhlhe41	Basic helix-loop-helix family, member e41	1.320,164,851	1.48E-19	2.54E-17
73,442	Hspa12a	Heat shock protein 12 A	1.420,015,579	3.70E-19	6.16E-17
66,203	Lce1m	Late cornified envelope 1 M	2.169,646,121	6.75E-19	1.07E-16
435,350	Serpinb6e	Serine (or cysteine) peptidase inhibitor, clade B, member 6e	3.976,679,949	4.75E-18	6.83E-16
69,117	Adh6a	Alcohol dehydrogenase 6 A (class V)	2.187,395,639	1.07E-17	1.48E-15
238,395	Serpina3j	Serine (or cysteine) peptidase inhibitor, clade a (alpha-1 antiproteinase, antitrypsin), member 3 J	2.590,357,248	1.13E-17	1.56E-15
107,585	Dio3	Deiodinase, iodothyronine type III	1.781,280,765	1.45E-17	1.95E-15
11,519	Add2	Adducin 2 (beta)	1.861,512,402	2.43E-17	3.14E-15
271,047	Serpina3b	Serine (or cysteine) peptidase inhibitor, clade A, member 3 B	3.973,024,928	7.68E-17	9.65E-15
12,411	Cbs	Cystathionine beta-synthase	1.100,796,697	8.20E-17	1.02E-14
68,659	Fam198 b	Family with sequence similarity 198, member B	1.064,341,173	3.47E-16	3.95E-14
192,166	Sardh	Sarcosine dehydrogenase	1.064,351,251	5.48E-16	6.04E-14
18,162	Npr3	Natriuretic peptide receptor 3	1.39,877,771	2.50E-15	2.59E-13
Top 20 downregulated genes					
16,409	Itgam	Integrin alpha M	−2.359,917,295	1.99E-94	3.57E-90
170,677	Cdhr1	Cadherin-related family member 1	−3.677,378,859	1.32E-81	1.19E-77
16,414	Itgb2	Integrin beta 2	−2.253,358,932	1.70E-80	1.02E-76
19,354	Rac2	RAS-related C3 botulinum substrate 2	−2.283,072,327	1.24E-77	5.58E-74
83,382	Siglece	Sialic acid binding ig-like lectin E	−2.712,092,471	6.31E-76	2.27E-72
217,306	Cd300e	CD300 E molecule	−2.896,226,408	2.08E-73	6.24E-70
73,656	Ms4a6c	Membrane-spanning 4-domains, subfamily A, member 6C	−1.87,982,465	1.47E-54	3.76E-51
100,042,514	Sprr2a3	Small proline-rich protein 2A3	−4.039,888,592	1.45E-53	3.25E-50
83,490	Pik3ap1	Phosphoinositide-3-kinase adaptor protein 1	−1.852,487,496	1.62E-52	3.23E-49
15,163	Hcls1	Hematopoietic cell specific lyn substrate 1	−1.807,265,374	2.60E-47	4.68E-44
23,880	Fyb	FYN binding protein	−1.927,183,842	5.81E-45	9.50E-42
72,042	Cotl1	Coactosin-like 1 (dictyostelium)	−1.563,171,219	1.30E-44	1.94E-41
246,256	Fcgr4	Fc receptor, IgG, low affinity IV	−2.70,846,219	1.04E-43	1.44E-40
18,173	Slc11a1	Solute carrier family 11 (proton-coupled divalent metal ion transporters), member 1	−1.951,701,945	3.15E-43	4.05E-40
26,888	Clec4a2	C-type lectin domain family 4, member a2	−1.930,263,987	1.02E-41	1.22E-38
65,221	Slc15a3	Solute carrier family 15, member 3	−2.280,078,606	2.26E-41	2.54E-38
14,127	Fcer1g	Fc receptor, IgE, high affinity I, gamma polypeptide	−1.713,529,716	2.48E-41	2.62E-38
244,233	Cd163l1	CD163 molecule-like 1	−2.148,022,202	4.31E-41	4.31E-38
20,525	Slc2a1	Solute carrier family 2 (facilitated glucose transporter), member 1	−1.727,582,584	4.31E-40	4.08E-37
11,433	Acp5	Acid phosphatase 5, tartrate resistant	−1.490,765,474	6.27E-40	5.63E-37

Q-VALUE, A Benjamini value (an adjusted *p*-value).

The biological characteristics of the potential targets of TDGs were evaluated by KEGG and GO analyses. KEGG analysis results (Figure 3B) demonstrated that the most upregulated gene categories were for the neuroactive ligand-receptor interaction, estrogen signaling pathway, biosynthesis of unsaturated fatty acids, and glycine, serine, and threonine (Gly-Ser-Thr) metabolism. The most downregulated gene categories were cytokine-cytokine receptor interaction, *Staphylococcus aureus* infection, osteoclast differentiation, and chemokine signaling pathway. GO analysis results (Figure 3C) revealed that the most upregulated gene categories were intermediate filaments, keratin filaments, cellular components, and structural molecule activity. The most downregulated gene categories were inflammatory response, immune system process, extracellular space, and innate immune response. Thus, the above results might reflect abnormalities in biological processes, metabolism, and inflammatory immune-related signaling pathways in psoriasis, and manifest the conceivable mechanism of TDGs.

### Experimental Verification

#### Taodan Granules Increased the Gly-Ser-Thr Metabolism Axis and Decreased Chemokine Signaling Pathway and Inflammatory Marker Protein Expression

**FIGURE 4:**
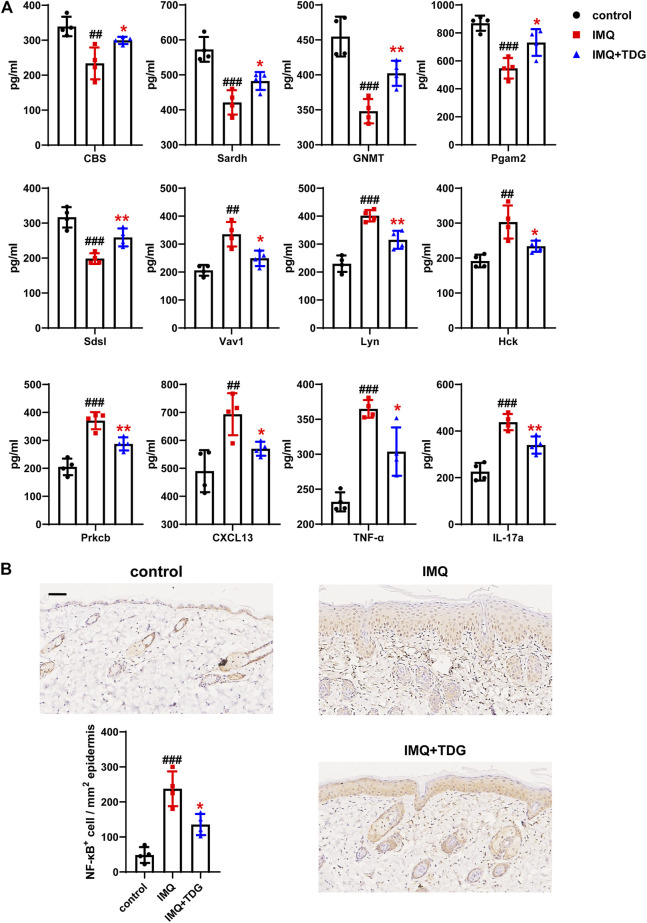
Increased gene expression in Gly**-**Ser-Thr metabolism axis and decreased gene expression in chemokine signaling pathway as well as inflammatory markers after Taodan granule (TDG) treatment. **(A)** Expression determined by ELISA: CBS, Sardh, GNMT, Pgam2, and Sdsl in Gly-Ser-Thr axis; Vav1, Lyn, Hck, Prkcb, and CXCL13 in chemokine signaling pathway; IL-17a as well as TNF-α protein in lesions. **(B)** Representative immunohistochemistry sections of NF-κB nuclear staining (brown) of the back skin lesions (200×). Quantification of NF-κB^+^ cells in back lesions. Scale bar = 100 μm. The data are expressed as the mean ± SD. Four skin lesions in each group were included for analysis. ^#^ *p* < 0.05, ^##^ *p* < 0.01, ^###^ *p* < 0.001, compared with the control group. *p < 0.05, **p < 0.01, ***p < 0.001, compared with the imiquimod (IMQ) group.

Several studies (Gelfand et al., 2018; Tollefson et al., 2018; Evans et al., 2020) have shown that the pathogenesis of psoriasis is closely related to metabolic disorders that are frequently aggravated. The Gly-Ser-Thr axis is a major metabolic crossroad connecting several crucial biological pathways (Aon et al., 2020). Based on the sequencing results, the genes of CBS, Sardh, GNMT, Pgam2, and Sdsl in the Gly-Ser-Thr axis were selected for the determination of protein concentrations on day 12 to explore the involvement of this metabolic pathway in the TDG treatment of psoriasis (Figure 4A). Compared with the IMQ group, the skin protein expression of these five genes in the IMQ + TDG group was significantly increased (CBS: *p* = 0.030; Sardh: *p* = 0.030; GNMT: *p* = 0.005; Pgam2: *p* = 0.022; Sdsl: *p* = 0.007), which was consistent with our KEGG enrichment results (Figure 3B).

Furthermore, the chemokine signaling pathway, which controls inflammatory response and directional cell migration, is involved in KC hyperplasia, regulating inflammation, the formation of new blood vessels, and other processes linked to psoriasis-related damage. KEGG analysis revealed that the chemokine signaling pathway was downregulated by TDG treatment (Figure 3B). Next, we validated the skin protein expression levels of the genes (Vav1, Lyn, Hck, Prkcb, and CXCL13) in this pathway on day 12, and a decrease in the same expression as KEGG was observed (Vav1: *p* = 0.016; Lyn: *p* = 0.004; Hck: *p* = 0.032; Prkcb: *p* = 0.005; CXCL13: *p* = 0.020) (Figure 4A).

In addition, compared with the IMQ group, the protein expression of IL-17a (*p* = 0.008) and TNF-α (*p* = 0.016) in the back lesions decreased in the IMQ + TDG group on day 12 (Figure 4A), which are inflammatory factors involved in the pathogenesis of psoriasis (Hawkes et al., 2017). We also examined NF-κB in a protein complex that controls the transcriptional regulation of inflammatory cytokines. Previous studies have shown that NF-κB is activated during psoriasis (Capon et al., 2012). Here, the downregulation of NF-κB by TDGs was observed (back: *p* = 0.013; ear: *p* = 0.027) (Figure 4B; Supplementary Figure S5). Consistent with the above results, KEGG analysis showed TNF-α together with IL-17a and NF-κB-related pathways were downregulated by TDG treatment (Figure 3B).

#### Taodan Granules Reduced Protein Expressed Levels of Rac2 in Psoriatic Lesions and Serum Arhgdib

**FIGURE 5:**
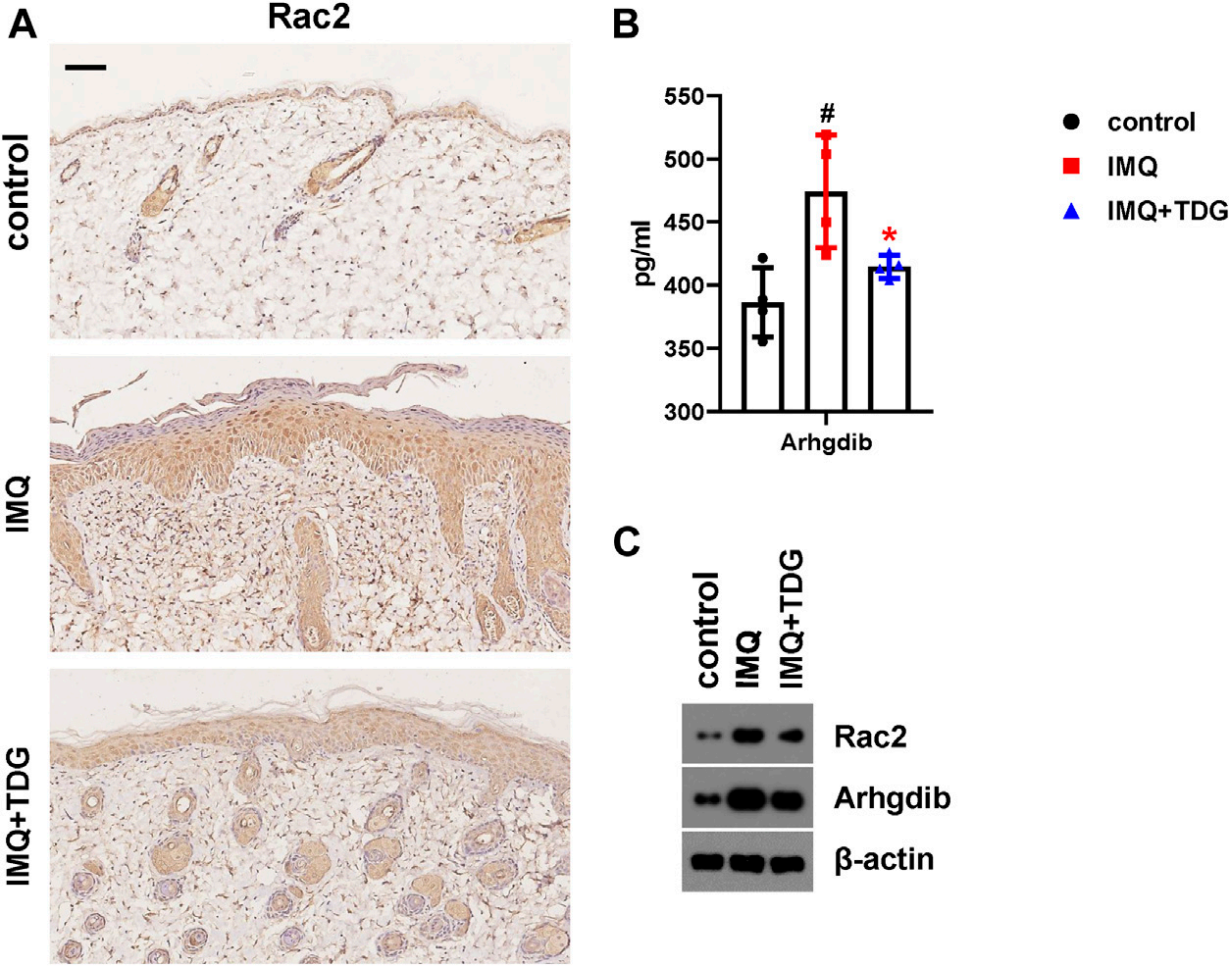
Reduced Rac2 and Arhgdib in lesions following Taodan granule (TDG) treatment. **(A)** Representative images of Rac2 cytoplasm staining (brown) of back lesions from each group (200×). Scale bar = 100 μm. **(B)** The expression of Arhgdib protein in lesions determined by ELISA. **(C)** Western blotting of Rac2 and Arhgdib protein in back lesions on day 12. The data are expressed as mean ± SD. Four skin lesions in each group were included for analysis. ^#^ *p* < 0.05, ^##^ *p* < 0.01, ^###^ *p* < 0.001, compared with the control group. *p < 0.05, **p < 0.01, ***p < 0.001, compared with the imiquimod (IMQ) group.

Abnormal proliferation and migration of KCs in psoriasis are vital pathogenic factors (Zhang et al., 2019). Rac2, a member of the small Rho GTPase family, centrally regulating cell migration via cytoskeletal rearrangement (Hordijk, 2006). Our mRNA sequencing results demonstrated that Rac2 was significantly downregulated following TDG treatment (Table 1). To verify whether TDGs could decrease the expression of Rac2, which influenced the regulation of migration in IMQ-induced psoriasis-like mice, IHC was used to detect Rac2 protein expression on day 12. The TDG-treated lesions demonstrated a significant decrease in the number of Rac2-positive cells (Figure 5A; Supplementary Figure S5) in the basal layers of the *epidermis* compared to the IMQ group. The same results were found on western blotting to verify back lesions (Figure 5C).

To further explore the feasible mechanism by which TDGs regulate Rac2 in psoriasis, we conducted a protein-protein interaction analysis using the Search Tool for the Retrieval of Interaction Gene/Proteins (String) database based on our sequencing results (Table 2). The results showed that the Arhgdib, Ncf2, and Cybb genes in DEGs had the highest combined scores for Rac2. We chose to assess the serum expression of the Arhgdib gene, a Rho GDI protein, to test the possible mode action of Rac2 during TDG treatment of psoriasis on day 12. Following TDG treatment, the protein expression of Arhgdib in IMQ-induced psoriasis-like mice was significantly downregulated (*p* = 0.040) (Figure 5B), which matched our sequencing results. Simultaneously, we also affirmed the protein expression of Arhgdib in the back lesions through western blotting, consistent with the trend of expression by ELISA (Figure 5C). These results suggest that TDGs may alleviate the symptoms of psoriasis by regulating Rac2 and Arhgdib, which are related to the mechanism of cell migration.

**TABLE 2:** Top 20 genes of combined-score with Rac2 (Genes with |log2FoldChange| > 1 and *p*-value < 0.05).

Gene ID	Gene name	Gene definition	LOG_2_∣FC∣	*p*-value	*Q*-value	Regulation	Combined-score
11,857	Arhgdib	Rho, GDP dissociation inhibitor (GDI) beta	−1.363,083,474	9.26E-34	4.62E-31	DOWN	994
17,970	Ncf2	Neutrophil cytosolic factor 2	−1.116,136,949	2.99E-14	2.68E-12	DOWN	991
13,058	Cybb	Cytochrome b-245, beta polypeptide	−1.010,635,235	4.13E-08	1.15E-06	DOWN	989
17,972	Ncf4	Neutrophil cytosolic factor 4	−1.477,689,282	5.77E-24	1.62E-21	DOWN	988
22,324	Vav1	Vav 1 oncogene	−1.810,926,467	1.18E-37	8.16E-35	DOWN	987
105,855	Nckap1l	NCK associated protein 1 like	−1.131,010,001	1.11E-21	2.37E-19	DOWN	981
17,969	Ncf1	Neutrophil cytosolic factor 1	−1.299,760,797	1.81E-21	3.70E-19	DOWN	977
277,360	Prex1	Phosphatidylinositol-3,4,5-trisphosphate-dependent rac exchange factor 1	−1.038,248,484	2.29E-19	3.88E-17	DOWN	967
94,176	Dock2	Dedicator of cyto-kinesis 2	−1.092,696,865	1.23E-17	1.67E-15	DOWN	963
171,207	Arhgap4	Rho gtpase activating protein 4	−1.592,067,061	8.27E-28	2.86E-25	DOWN	941
18,707	Pik3cd	Phosphatidylinositol 3-kinase catalytic delta polypeptide	−1.016,119,488	1.31E-14	1.24E-12	DOWN	909
223,881	Rnd1	Rho family gtpase 1	−1.900,093,986	0.000995	0.007526	DOWN	906
23,912	Rhof	ras homolog family member F (in filopodia)	−1.620,812,138	0.000323	0.002942	DOWN	906
320,207	Pik3r5	Phosphoinositide-3-kinase, regulatory subunit 5, p101	−1.91,468,164	9.01E-19	1.42E-16	DOWN	821
18,751	Prkcb	Protein kinase C, beta	−1.350,798,593	1.38E-27	4.66E-25	DOWN	806
15,163	Hcls1	Hematopoietic cell specific lyn substrate 1	−1.807,265,374	2.60E-47	4.68E-44	DOWN	787
22,376	Was	Wiskott-aldrich syndrome	−1.152,788,414	2.68E-09	1.01E-07	DOWN	772
19,264	Ptprc	Protein tyrosine phosphatase, receptor type, C	−1.165,374,311	4.22E-14	3.70E-12	DOWN	772
16,414	Itgb2	Integrin beta 2	−2.253,358,932	1.70E-80	1.02E-76	DOWN	756
107,321	Lpxn	Leupaxin	−1.469,153,083	6.09E-09	2.08E-07	DOWN	751

Q-VALUE, A Benjamini value (an adjusted *p*-value).

## Discussion

TDGs have therapeutic effects in relieving psoriasis. Our previous studies have shown that TDGs are effective for the treatment of mild-to-moderate psoriasis vulgaris (Fan et al., 2006a; Fan et al., 2006b), although the mechanism remains unclear. CM mainly exerts therapeutic effects via the interaction of multiple natural products containing an army of targets through numerous pathways, which is a challenge for investigating their mechanisms of action (Zhou et al., 2014). Nonetheless, RNA-seq may provide a new direction for this conundrum. In this study, we performed RNA-seq to determine the mechanism by which TDGs ameliorate psoriasis and verify the observations via *in vivo* experiments.

To clarify the pharmacological mechanism of TDGs, we used HPLC to establish quality control. Based on the excellent curative effect and unclear mechanism of TDGs in this study, we first induced a mouse psoriatic model with IMQ, which is a classic accepted model of psoriasis that we have often used in our previous studies, to verify the effectiveness of TDGs (Kuai et al., 2020; Ru et al., 2020). Our results demonstrated that TDGs attenuated IMQ-induced psoriatic classical symptoms (Figure 2; Supplementary Figure. S1). To assess whether the remission of psoriatic lesions after TDG treatment may be due to the prevention of the over-proliferation of KCs, we verified the Ki67 and PCNA-positive cells in each group. Ki67 and PCNA are classical markers of cell proliferation in a variety of diseases (Alhosaini et al., 2021; Cai et al., 2021). As expected, following TDG treatment, the number of Ki67 and PCNA-positive cells in IMQ-induced psoriasis-like mice decreased significantly (Figure 2; Supplementary Figure S1), which is consistent with the results of previous studies (Ru et al., 2020; Thatikonda et al., 2020; Tomalin et al., 2020). Accordingly, we speculated that TDGs could mitigate the excessive proliferation of KCs to effectively alleviate psoriasis.

To further explore the potential mechanism of TDG in psoriasis, we applied RNA-seq to investigate skin lesion samples from IMQ-induced psoriasis-like mice with and without TDG. A total of 1,233 DEGs were identified, of which 539 were upregulated and 694 were downregulated (Figure 3A). The mRNA levels of the 10 most significantly upregulated and downregulated DEGs were verified by RT-PCR, consistent with the sequencing results (Supplementary Figure S4).

A bioinformatic analysis concluded that the most upregulated gene categories by TDGs were associated with metabolism-related signaling pathways (Figures 3B,C); these pathways have been found to mediate psoriasis in several existing reports (Chen et al., 2018; Lin et al., 2019). The Gly-Ser-Thr axis is a metabolic pathway closely related to proliferative disease and lifetime (Aon et al., 2020). Nevertheless, no related studies on this axis in psoriasis have been reported. RNA-seq results confirmed that CBS, Sardh, GNMT, Pgam2, and Sdsl were the top five upregulated genes by TDGs in the Gly-Ser-Thr axis. CBS is primarily a cytosolic enzyme that regulates homocysteine metabolism and is involved in the biosynthesis of hydrogen sulfide. Clinical evidence has strongly supported the negative regulatory role of CBS in proliferative diseases (Kim et al., 2009), which induces autophagy and apoptosis via the PI3K/Akt/mTOR pathway in hepatocellular carcinoma (HCC) cells (Zhu et al., 2018). Sardh and GNMT are pivotal enzymes associated with sarcosine metabolism, and transcription and protein levels are regulated in cancer tissues. Meanwhile, Sardh has been reported to reduce sarcosine levels and attenuate the invasion of DU145 prostate cancer cells (Khan et al., 2013). Pgam2 is a crucial enzyme involved in glycolysis related to oxidative stress, and decreased expression of Pgam2 was observed in proliferative disease induced by local infections (Cladel et al., 2019). In addition, clinical evidence has suggested that Sdsl has specific protein expression in peripheral cholangiocarcinoma (Darby et al., 2010). We verified the upregulation of CBS, Sardh, GNMT, Pgam2, and Sdsl following TDG treatment (Figure 4A).

Moreover, the most downregulated gene categories by TDGs were for the canonical immune and inflammatory pathways associated with psoriasis (Figures 3B,C). RNA-seq reported that Vav1, Lyn, Hck, Prkcb, and CXCL13 were the top five downregulated genes in the chemokine signaling pathway. Vav1, a key downstream signaling molecule of the T-cell receptor, can trigger cytoskeleton rearrangement and immune synapse formation, as well as lead to the activation of transcription factors along with cytokine release (NFAT, NF-κB, AP1, etc.) (Bustelo, 2000). During the development of a host of tumor diseases, Vav1 promotes cell proliferation and invasion (Fernandez-Zapico et al., 2005). Lyn is the substrate for caspases in the cysteine protease family and is involved in the regulation of apoptosis and inflammation. Lyn has been validated for specific increases in protein levels in human psoriatic lesions and is involved in regulating the expression of STAT3 and the NF-κB pathway to mediate a chronic inflammatory syndrome resembling human psoriasis in a mouse model by pan-genomic profiling (Marchetti et al., 2009). Hck, a member of the Src family of kinases, is highly expressed in macrophages and is related to various inflammatory responses (Kong et al., 2020). Clinical evidence has shown that the mRNA level of Prkcb in the bone marrow is significantly higher in patients with psoriasis than in healthy individuals. Prkcb is a critical gene for regulating hematopoietic cell development and differentiation, and the abnormal expression of Prkcb may induce dysfunction in the hematopoietic cells of patients with psoriasis (Yin et al., 2011). The inflammatory chemokine CXCL13 is crucial in the homing of B lymphocytes into lymphoid follicles, while CXCL13 mRNA was upregulated in the brain tissues of the IMQ-induced mouse model (McColl et al., 2016). By verifying the sequencing results via ELISA, we found that TDGs could downregulate the expression of these five genes (Figure 4A), and the specific mechanism is worthy of further exploration.

Psoriasis has been identified as a T-cell-mediated autoimmune skin disease. Different T-cell subsets such as Th1, Th17, and regulatory T cells are pivotal for psoriatic pathogenesis and inflammation (Armstrong et al., 2020). The psoriatic inflammatory cascade is triggered by plasmacytoid dendritic cells and activated and polarized Th cells, while producing various inflammatory cytokines (IL-23, IL-6, IFN-γ, IL-17a, TNF-α, etc.) (Zhu et al., 2006; Lowes et al., 2013; Wu et al., 2018). These pro-inflammatory factors induce epidermal over-proliferation and activate KCs to produce chemokines and antimicrobial peptides to maintain the inflammatory microenvironment and promote the development of the psoriatic phenotype (Conrad et al., 2018). Our previous studies have demonstrated that the expression levels of IL-23, IL-17, IFN-γ, and TNF-α in the serum of patients with psoriasis were significantly increased (Li et al., 2016). Furthermore, NF-κB is a crucial regulator of inflammation, cell proliferation, differentiation, and apoptosis in the pathogenesis of psoriasis. The NF-κB pathway regulates the expression of pro-inflammatory factors (TNF-α and IL-6) to aggravate the inflammatory process. IL-6 induces IL-17 to produce an inflammatory response, promotes KC hyperproliferation, and increases T-cell aggregation in the *epidermis*. IL-17 and TNF-α promote the expression of CCL20 in KCs and further attract dendritic cells as well as Th17 cells, thereby promoting the formation of chemotaxis rings and exacerbating the inflammatory response in psoriasis (Wang et al., 2015; Yang et al., 2020). Several *in vivo* experiments have also indicated that the inhibition of NF-κB activity could significantly improve the inflammatory response in psoriasis (Kulkarni et al., 2015; Irrera et al., 2017). To further verify whether TDGs could regulate skin inflammation, we measured the concentrations of IL-17a, TNF-α, and NF-κB proteins in each group. Consistent with previous results (Rerknimitr et al., 2016; Yu et al., 2019), the expression of IL-17a, TNF-α, and NF-κB was significantly reduced after TDG treatment (Figure 4; Supplementary Figure S5). Based on the above, we inferred that TDGs upregulated metabolic pathways, such as the Gly-Ser-Thr axis, and downregulated immune- and inflammation-related signaling pathways to treat psoriasis.

Rac2, a major isoform of the Rac GTPases, has been shown to play a crucial role in cell migration and is involved in the homing of T-lymphoid progenitor cells (Lu et al., 2020). High expression of Rac2 may be a diagnostic index for clear-cell renal cell carcinoma, as the knockdown of Rac2 *in vitro* attenuates the proliferation, migration, and invasion of renal carcinoma cells (Liu et al., 2019). The hyperproliferation and abnormal migration of KCs, which are responsible for psoriasis-lesioned microenvironments, are critical features of psoriasis (Liu et al., 2020). Our sequencing results demonstrated the downregulation of Rac2 in skin lesions of IMQ-induced psoriasis-like mice by TDGs (Table 1). Therefore, IHC and western blotting were used to confirm that Rac2 expression significantly decreased in skin lesions after TDG treatment, to verify the feasible role of TDGs in regulating Rac2 (Figure 5A; Supplementary Figure S5). Next, based on the protein-protein interaction analysis, we found that the skin protein expression of Arhgdib (had the highest combined scores of Rac2) in IMQ-induced psoriasis-like mice was significantly downregulated (Figure 5B). Arhgdib is mainly located in hematopoietic, endothelial, and epithelial cells. Clinical evidence has demonstrated that Arhgdib is often highly expressed in proliferative diseases, and knockout-Arhgdib can reduce proliferation, migration, and invasion of cells *in vitro* (Wang et al., 2020). Hence, we speculate that TDGs may reduce Rac2 and Arhgdib and, consequently, decrease KC proliferation and migration to relieve psoriasis.

## Conclusion

We explored the feasible mechanism of action of TDG treatment for psoriasis through RNA-seq analysis and experimental verification. We found that TDGs affected KC proliferation and inflammatory responses to alleviate IMQ-induced psoriatic symptoms. The mechanisms of TDG treatment for psoriasis include the upregulation of metabolic signaling pathways, downregulation of immune and inflammatory pathways, and a decrease in Rac2 and Arhgdib genes. Further studies are needed to clarify the mechanisms of TDGs and provide a reference for clinical indications, along with optimal doses. The fuzziness of Chinese herbal compounds is significant; hence, evidence of their effectiveness *in vitro* and *in vivo* is the foundation for all subsequent studies. In future studies, we plan to continue further exploring other signaling pathways involved.

## Data Availability

The data generated from this article can be found in the Sequence Read Archive database (https://www.ncbi.nlm.nih.gov/sra/), using accession number SRP292449.
