# Supplementary material for: Transcriptomic Analysis of the Mechanisms for Alleviating Psoriatic Dermatitis Using Taodan Granules in an Imiquimod-Induced Psoriasis-like Mouse Model
Source: Front Pharmacol. 2021 Apr 14;12:632414. doi: 10.3389/fphar.2021.632414 (PMC8114823; doi:10.3389/fphar.2021.632414)
Supplement: Supplementary file 1 [file Image1.tiff]

Frontiers | Transcriptomic analysis of the mechanisms for alleviating psoriatic dermatitis using Taodan granules in an imiquimod-induced psoriasis-like mouse model | Pharmacology


- About
- Journals
- Research Topics
- Articles
- More

Submit

My Frontiers

Office

- TSOF
  - TSOF
  - Article Production

Typesetter 3

frontiersproduction@tnq.co.in

- Profile
- Settings & Privacy
- Help Center
- Logout

Submit

**Impact Factor 4.225** | **CiteScore 5.0**More on impact ›

|  |  |
| --- | --- |
| Frontiers in Pharmacology | Ethnopharmacology |

Toggle navigation


Section


- (current)Section
- About
- Articles
- Research topics
- For authors 
  - Why submit?
  - Fees
  - Article types
  - Author guidelines
  - Review guidelines
  - Submission checklist
  - Contact editorial office
  - Submit your manuscript
- Editorial board

- *Article alerts*

##### This article is part of the Research Topic

Targeting Human Inflammatory Skin Diseases with Natural Products: Exploring Potential Mechanisms and Regulatory Pathways
View all
9
Articles

Articles


**Suggest a Research Topic >**

- 59
  total views

 View Article Impact

**Suggest a Research Topic >**

##### SHARE ON

- Facebook

  0
- Twitter

  0
- LinkedIn

  0
- AddThis

  New


## Original Research ARTICLE

Front. Pharmacol.
| doi: 10.3389/fphar.2021.632414

# Transcriptomic analysis of the mechanisms for alleviating psoriatic dermatitis using Taodan granules in an imiquimod-induced psoriasis-like mouse model Provisionally accepted The final, formatted version of the article will be published soon. **Notify me**

Le Kuai1,  Ying Luo1,  Keshen Qu2,  Yi Ru1,  Yue Luo1,  Xiaojie Ding1,  Meng Xing1,  Liu Liu1,  Xiaoying Sun1,  Xin Li1 and  Bin Li1\*

- 1Yueyang Hospital of Integrated Traditional Chinese and Western Medicine, Shanghai University of Traditional Chinese Medicine, China
- 2Longhua Hospital, Shanghai University of Traditional Chinese Medicine, China

Taodan granules (TDGs) are clinically efficacious for treating psoriasis, buttheir specific mechanisms of action are unclear. In this study, we determined the concentrations of tanshinone IIA and curcumol using high-performance liquid chromatography (HPLC) to establish quality control parameters for assessing the mechanism of TDGs in treating psoriasis. Thereafter, a mouse model of psoriasis was treated with TDGs. TDGs attenuated imiquimod-induced typical erythema, scales, and thickening of the back and ear lesions in the psoriatic mouse model. Furthermore, PCNA- and Ki67-positive cells were reduced in the epidermis of psoriatic lesions following TDG treatment. Finally, the sequencing results were verified using a multitude of methods, and the mechanism of action of TDGs against psoriasis was found to be via the upregulation of metabolic signaling pathways such as the Gly Ser-Thr axis, the downregulation of immune and inflammatory pathways, and the decrease in Rac2 and Arhgdib concentrations. Overall, this study clarified the mechanism of TDG treatment for psoriasis and provided evidence for its clinical application.

Keywords: 
Psoriasis, Taodan granules, RNA sequencing analysis, Chinese medicine, Transcriptomic Analysis

Received: 23 Nov 2020;
Accepted: 11 Mar 2021.

Copyright: © 2021 Kuai, Luo, Qu, Ru, Luo, Ding, Xing, Liu, Sun, Li and Li. This is an open-access article distributed under the terms of the Creative Commons Attribution License (CC BY). The use, distribution or reproduction in other forums is permitted, provided the original author(s) and the copyright owner(s) are credited and that the original publication in this journal is cited, in accordance with accepted academic practice. No use, distribution or reproduction is permitted which does not comply with these terms.

\* Correspondence: 
Prof. Bin Li, Yueyang Hospital of Integrated Traditional Chinese and Western Medicine, Shanghai University of Traditional Chinese Medicine, Shanghai, 200437, China, 18930568129@163.com

Write a comment...

Add

##### COMMENTARY

##### ORIGINAL ARTICLE

##### People also looked at

## Chinese Medicine Formula Kai-Xin-San Ameliorates Neuronal Inflammation of CUMS-Induced Depression-like Mice and Reduces the Expressions of Inflammatory Factors via Inhibiting TLR4/IKK/NF-κB Pathways on BV2 Cells

Suchen Qu, Mengqiu Liu, Cheng Cao, Chongqi Wei, Xue-Er Meng, Qianyin Lou, Bin Wang, Xuan Li, Yuyan She, Qingqing Wang, Zhichao Song, Zhengxiang Han, Yue Zhu, Fei Huang and Jin-Ao Duan

## Genetic Approaches for the Treatment of Facioscapulohumeral Muscular Dystrophy

Kenji Rowel Q. Lim and Toshifumi Yokota

## Leflunomide induces dose-dependent lung injury in mice via stimulating vimentin and NLRP3 inflammasome production

Mohamed El-Sherbiny, Hoda Atef, Mohamed Ahmed Eladl, Abdelaty Shawky Mohamed, Mohamed El-Shafey, Howaida S. Ali, Sawsan A. Zaitone, Suliman Y. Alomar, Saeed Awad M. Alqahtani, Sheka Yagub Aloyouni and Mohammed A Attia

**Suggest a Research Topic >**

×

#### Supplementary Material

  

There is no supplementary material currently available for this article

Loading supplemental data...

  

|  | File Name |  |
| --- | --- | --- |
|  | Table 1.DOCX |  |
|  | Table 2.DOCX |  |
|  | Image 1.TIFF |  |
|  | Image 2.TIFF |  |
|  | Image 3.TIFF |  |
|  | Image 4.TIFF |  |
|  | Image 5.TIFF |  |

  

Close

- About Frontiers
- Institutional Membership
- Books
- News
- Frontiers' social media
- Contact
- Careers
- Submit
- Newsletter
- Help Center
- Terms & Conditions
- Privacy Policy

© 2007 - 2021 Frontiers Media S.A. All Rights Reserved

### Privacy Preference Center

Our website uses cookies that are necessary for its operation. Additional cookies are only used with your consent. These cookies are used to store and access information such as the characteristics of your device as well as certain personal data (IP address, navigation usage, geolocation data) and we process them to analyse the traffic on our website in order to provide you a better user experience, evaluate the efficiency of our communications and to personalise content to your interests. Some cookies are placed by third-party companies with which we work to deliver relevant ads on social media and the internet. Click on the different categories' headings to change your cookie preferences. Click on "More Information" if you wish to learn more about how data is collected and shared.
More information

### Manage Consent Preferences

#### Strictly Necessary Cookies

Always Active

These cookies are necessary for the website to function and cannot be switched off in our systems. They are usually only set in response to actions made by you which amount to a request for services, such as setting your privacy preferences, logging in or filling in forms. You can set your browser to block or alert you about these cookies, but some parts of the site will not then work. These cookies do not store any personally identifiable information.

#### Analytics Cookies

Analytics Cookies

These cookies allow us to count visits and traffic sources so we can measure and improve the performance of our site. They help us analyse which pages are the most and least popular and see how visitors move around the site.    All information these cookies collect is aggregated and therefore anonymous.

#### Functional Cookies

Functional Cookies

These cookies enable the website to provide enhanced functionality and personalisation. They may be set by us or by third party providers whose services we have added to our pages. If you do not allow these cookies then some or all of these services may not function properly.

#### Advertising Cookies

Advertising Cookies

These cookies may be set through our site by our advertising partners. They may be used by those companies to build a profile of your interests and show you relevant adverts on other sites.    They do not store directly personal information, but are based on uniquely identifying your browser and internet device. If you do not allow these cookies, you will experience less targeted advertising.

### Back Button Performance Cookies

Vendor Search  Search Icon

Filter Icon

Clear

checkbox label label

Apply Cancel

Consent Leg.Interest

checkbox label label

checkbox label label

checkbox label label

Confirm My Choices
