# Supplementary material for: Transcriptomic Analysis of the Mechanisms for Alleviating Psoriatic Dermatitis Using Taodan Granules in an Imiquimod-Induced Psoriasis-like Mouse Model
Source: Front Pharmacol. 2021 Apr 14;12:632414. doi: 10.3389/fphar.2021.632414 (PMC8114823; doi:10.3389/fphar.2021.632414)
Supplement: Supplementary file 6 [file table1.docx]

**Table S1** **Prescription of TDG**

| Main composition | English translation | Amount (g) | Lot No. | Commercial source |
| --- | --- | --- | --- | --- |
| *Salvia miltiorrhiza Bunge* | Danshen Root | 30 | 2004201-1 | Shanghai Wanshicheng National Pharmaceutical Products Co., LTD |
| *Curcuma aeruginosa Roxb.* | Zedoary | 30 | 200309 | Shanghai Hongqiao Traditional Chinese Medicine Decoction pieces Co., LTD |
| *Astragalus mongholicus Bunge* | Root | 15 | 20200519-1 | Shanghai Wanshicheng National Pharmaceutical Products Co., LTD |
| *Glycyrrhiza inflata Batalin* | Liquoric Root | 10 | 200426 | Shanghai Hongqiao Traditional Chinese Medicine Decoction pieces Co., LTD |
| *Angelica sinensis (Oliv.) Diels* | Chinese Angelica | 15 | 2020011002 | Shanghai Huapu Chinese Medicine Decoction pieces Co., LTD |
| *Conioselinum anthriscoides 'Chuanxiong'* | Szechuan Lovage Rhizome | 10 | 200409 | Shanghai Kangqiao Pharmaceutical Co., LTD |
| *Prunus persica (L.) Batsch* | Peach Seed | 10 | 2020021008 | Shanghai Huapu Chinese Medicine Decoction pieces Co., LTD |
| *Cyathula officinalis K.C.Kuan* | Medicinal Cyathula Root | 15 | 200226 | Shanghai Qingpu Traditional Chinese Medicine Yinpian Co., LTD |
| *Smilax china L.* | Chinaroot Greenbier Rhizome | 30 | 2020051201 | Shangyao Yutiancheng (Shanghai) Pharmaceutical Co., LTD |
